# Considerations for accurate gene expression measurement by reverse transcription quantitative PCR when analysing clinical samples

**DOI:** 10.1007/s00216-014-7857-x

**Published:** 2014-05-25

**Authors:** Rebecca Sanders, Deborah J. Mason, Carole A. Foy, Jim F. Huggett

**Affiliations:** 1 Present Address: Molecular & Cell Biology, LGC, Queens Road, Teddington, TW11 0LY UK; 2 School of Biosciences, Cardiff University, Sir Martin Evans Building, Museum Avenue, Cardiff, CF10 3AX UK

**Keywords:** RNA, Uncertainty, Normalisation, Reverse transcription quantitative PCR, Standardisation

## Abstract

Reverse transcription quantitative PCR is an established, simple and effective method for RNA measurement. However, technical standardisation challenges combined with frequent insufficient experimental detail render replication of many published findings challenging. Consequently, without adequate consideration of experimental standardisation, such findings may be sufficient for a given publication but cannot be translated to wider clinical application. This article builds on earlier standardisation work and the MIQE guidelines, discussing processes that need consideration for accurate, reproducible analysis when dealing with patient samples. By applying considerations common to the science of measurement (metrology), one can maximise the impact of gene expression studies, increasing the likelihood of their translation to clinical tools.

**Figure:**
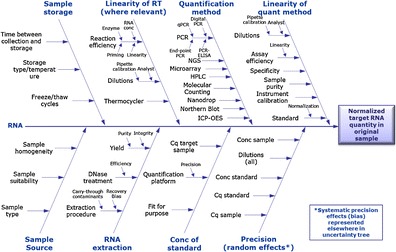
ᅟ

ᅟ

## Introduction

The real-time quantitative polymerase chain reaction (qPCR) [1], developed from the revolutionary method of polymerase chain reaction (PCR) pioneered by Kary Mullis in the 1980s [2–4], has emerged as a widely used method for biological investigation because it can detect and precisely quantify very small amounts of specific nucleic acid sequences. This is coupled to an inherent simplicity that makes qPCR assays straightforward to design and perform. The characterisation of gene expression patterns through quantification of messenger RNA (mRNA), by coupling reverse transcription with PCR, as a surrogate of cell metabolism is a major application of this technology. Reverse transcription qPCR (RT-qPCR) makes possible rapid and precise assessment of changes in gene expression as a result of physiology, pathophysiology or development [5]. However, for RNA analyses to be clinically informative, reliable measurements that are reproducible between laboratories are essential. As much as 30 % of the costs of medical care budgets are in measurements and tests related to diagnosis [6]. This necessitates sustained efforts to improve the reliability of such measurements and tests, which play a key role in the continual development of effective health care systems.

In research studies, RT-qPCR has been used to measure bacterial gene expression [7, 8] or RNA viral loads [9–12], to evaluate cancer status or to track disease progression and response to treatment [13–15]. As a consequence, this method is being applied to the discovery and development of putative biomarkers. An example of successful translation of an RT-qPCR method to the patient is the Oncotype Dx assay, which predicts the potential benefits of chemotherapy and the likelihood of cancer recurrence [16–19] and thus can be used to stratify patients to different treatment regimens [20]. Furthermore, viral load monitoring using RT-qPCR is now routine for a number of RNA viruses [21].

RT-qPCR is used extensively in clinical research investigating putative biomarkers for disease diagnosis as well as for predictive and prognostic monitoring. However, on review of the literature, articles published reporting RT-qPCR data frequently do not report all experimental details relating to RT-qPCR experiments. Fundamental experimental details are often omitted when reporting gene expression measurements, including information pertaining to RNA quality, the rationale for the choice of the normalisation strategy, the location of the amplicon or detailed descriptions of the reverse transcriptase and PCR assay conditions [22, 23].

To facilitate good repeatability (measurements made by the same operator or instrument, and under the same conditions over a short period of time) and reproducibility (measurements made by different operators or instruments, and/or under different conditions) [24], key aspects of RT-qPCR experimental processes need to be reported, as outlined in the MIQE (from ‘*m*inimum *i*nformation for publication of *q*uantitative real-time PCR *e*xperiments’) guidelines, which propose a minimum standard for the provision of information for publications reporting qPCR experiments [25]. These cover key aspects including sample acquisition, assay design and validation as well as details about data analysis, enabling other scientists to easily assess and, if necessary, repeat the experiment [25, 26]. This is fundamental if findings are to be corroborated, which is in turn crucial for the observation to be translated into a clinically useful tool.

RNA measurement on a complex biological sample (such as a tissue biopsy sample) requires a series of steps, each of which contributes error that is often severalfold greater than the difference in the mRNA to be measured. Consequently, determining differences in gene expression in real scenarios requires consideration of the sources of error and appropriate normalisation mechanisms to control for them.

Yet measurement claims of biologically significant gene expression differences are routinely made without apparent consideration (or reporting) of such technical factors [27]. Consequently, although often statistically significant, these results may not be due to the biological phenomenon under investigation and/or may not be reproducible. Without assessment and consideration of the technical variability introduced at each stage of the experimental process, findings may be of limited practical use in the clinic because they are difficult to reproduce.

## From sample to final result: a series of steps

**Fig. 1:**
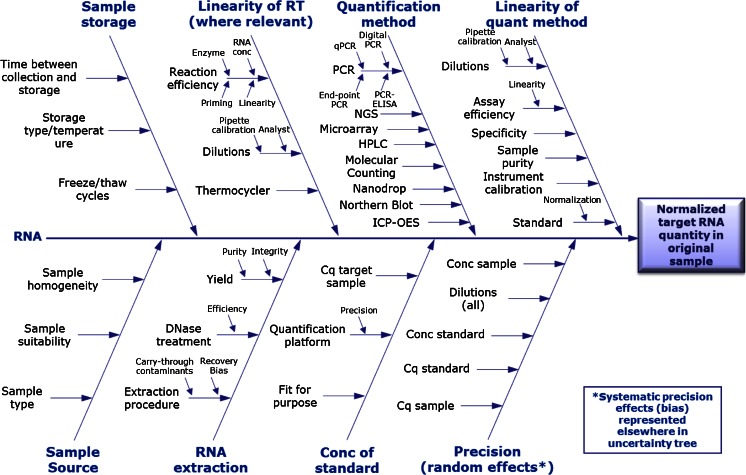
Cause and effect: uncertainty contributions for gene expression analysis. The *central arrow* represents the experimental process from RNA to the quantification method. Branches feeding into experimental progression characterise sources of variability that contribute to uncertainty at various stages of the process. There are numerous methods available for the final quantification step. *conc* concentration, *HPLC* high-performance liquid chromatography, *ICP-OES* inductively coupled plasma optical emission spectroscopy, *NGS* next-generation sequencing, *qPCR* quantitative PCR, *RT* reverse transcription

The route from sample to accurate quantification of gene expression is a multicomponent process, with each process having its own experimental uncertainty. There can be numerous factors that need to be considered (Fig. **1**). Cause-and-effect diagrams such as that in Fig. **1** are widely used in measurement uncertainty and the field of metrology [28, 29] to identify the relationships between sources of uncertainty. RT-qPCR techniques have the ability to quantify nucleic acids over a wide dynamic range (at least eight logarithms) and are precise (DNA and RNA measurements can typically be optimised to have a coefficient of variation of less than 5 % or less than 10 %, respectively [30]). But measurements using this precise technique are only as robust as the upstream processes used to sample, store and prepare the RNA. Precision is a measure of the degree of agreement between replicate measurement results obtained for the same sample [24, 31]. However, what is often overlooked is that the whole stepwise procedure contributes to the experimental precision.

It is well known that sample handling affects experimental variability [26], and source and storage conditions affect it too, all of which may contribute to variation in measurement, particularly if samples are obtained and analysed periodically during a successive long-term study. Consequently, sampling and subsequent storage should be carefully controlled and documented in order to preserve the quality and abundance of the RNA material.

**Fig. 2:**
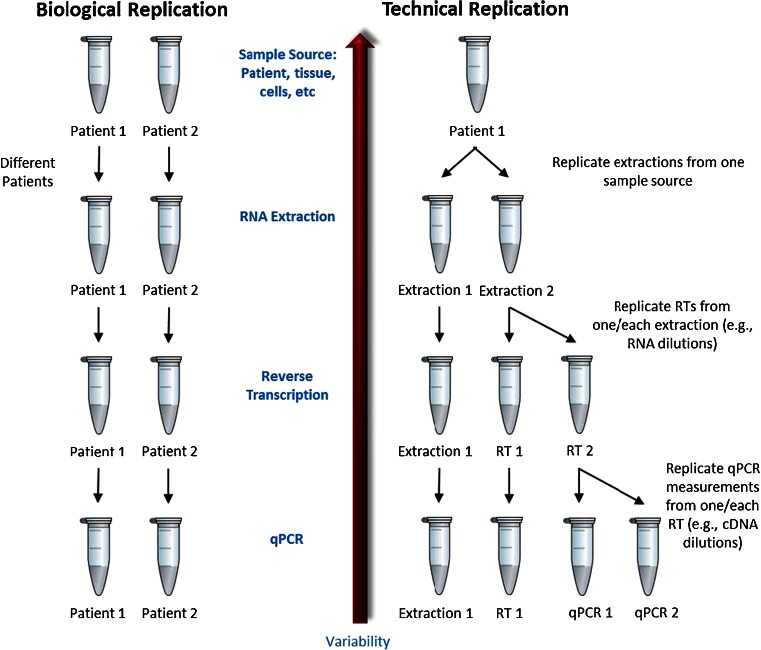
Different experimental designs representing biological versus technical replication. Generally, data variability increases as replication is included from higher stages within the experimental process. For example, to ascertain true patient variability, replicate biological samples must be analysed (different samples from one patient, samples from different tissues from the same patient, or samples from different patients). The RNA extraction and reverse transcription components of the process may contribute more variability to the final measurement than quantitative PCR (*qPCR*) alone. Definition of all sources of technical variability allows the actual biological variability to be discerned, and as such, more confidence can be conferred to the results when this variability is included. *cDNA* complementary DNA, *RT* reverse transcription

**Fig. 3:**
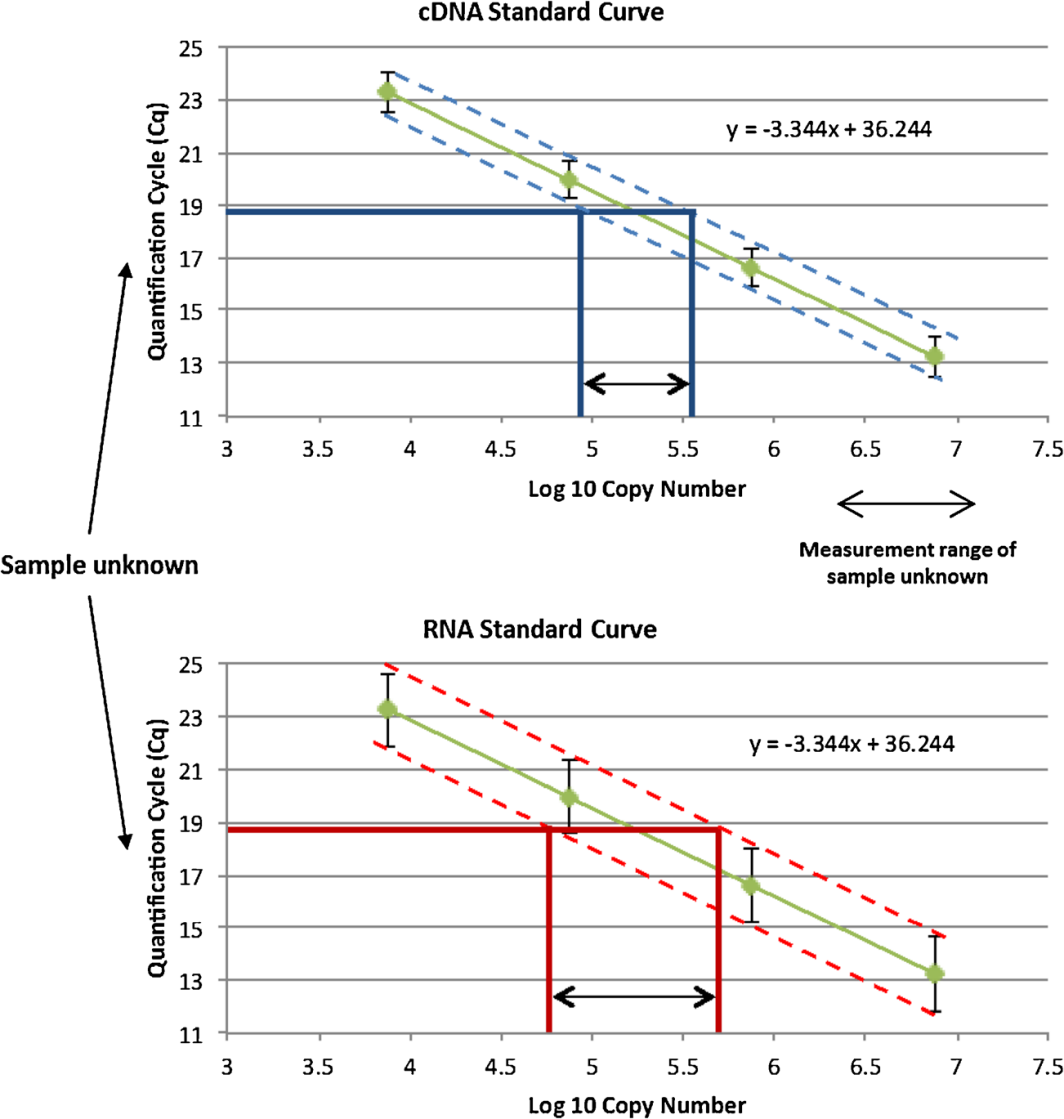
Variability observed between complementary DNA (*cDNA*) and RNA standard curves. The *green points* represent the standard curve. The variability of qPCR is relatively low when compared with reverse transcription variability. As a result, a standard curve generated from the dilution of cDNA indicates the variability associated with the qPCR step alone and does not represent variability associated with the reverse transcription step. Alternatively, a standard curve generated from an RNA dilution series incorporates the variability attributable to the reverse transcription step, which is intrinsically more variable than qPCR. Consequently, the range within which the unknown sample measurement can reliably lie is greater when using an RNA-based standard curve and smaller when using a DNA-based standard curve. The RNA curve will therefore provide a more accurate estimate of uncertainty, offering greater confidence in a result. Sample fold changes discerned when using this approach more likely represent ‘true’ measurement differences rather than insufficiently apportioned uncertainty

Both biological and technical replicates are recommended for good experimental design (Fig. **2**). Many studies have shown that variability attributed to reverse transcription is far greater than the variability contribution of qPCR alone [26, 32, 33] (Fig. **3**). This increased variance may be caused by factors such as reverse transcriptase efficiency, RNA integrity and secondary structure. The reverse transcription step is therefore critical for accurate RNA quantification. Reverse transcriptase linear dynamic range is another crucial consideration for successful RT-qPCR [32] and should be demonstrated empirically. However, often it is the PCR rather than the reverse transcription step that is replicated. This has the danger of appearing to produce highly precise data, but could in fact proffer bias by masking true measurement variability. Consequently, true, meaningful and clinically significant measurement, particularly of small expression fold changes, ideally requires a discussion of the potential different sources of variance and bias.

The use of distinct instruments, software, reagents, plates or seals can often lead to underestimated run-to-run differences that need to be compensated in order to allow data reproducibility [34]. Since there are so many steps involved in taking a tissue sample to a ‘quantitative’ result (Fig. **1**), it is not surprising that this variation is problematic [32], and factors that more comprehensively estimate error will lead to a better estimation of the variation and increase the likelihood of making accurate measurements.

Unexpected sources of RT-qPCR variability include the ability of the thermocycler to maintain a consistent temperature across all sample wells, as any deviations in temperature will lead to different reverse transcription and/or PCR amplification efficiencies [5, 35, 36] and thus contribute to the overall variability in measurement. This extends to differences between different thermocycler platforms, with differences observed in timing and heat transfer capabilities [36]. Expectation of lot-to-lot consistency may be a reason for selecting commercially available kits rather than preparing mixes in-house. In addition, maintenance of primer/probe stability is often assumed between different syntheses or suppliers. However, although the multitude of commercial kits and protocols available offer undeniable benefits, reagent preparations from distinct batches have been shown to contribute significant experimental variability, with up to sevenfold differences in calculated mRNA quantities observed [32, 37].

For some commercially supplied primers and probes, the location of the amplicon selected for gene expression is omitted, a fact that makes it difficult to adhere to the MIQE guidelines. The problem with not providing this information means the researcher does not know which part of a given transcript is being detected. This information is fundamental for any hope of reproducibility due to transcript differences, including alternative splicing, polyadenylation and alternative promoters. An amendment to the MIQE guidelines [38] offered a compromise to commercial vendors who do not disclose this information by alternatively requiring a context sequence to enable the researcher to locate which portion of a given sequence was being detected [25, 26, 38]. Where neither primer information nor a context sequence is provided, researchers using such commercial assays are strongly advised to sequence the PCR products to obtain the location of the transcript being measured.

The issues described above highlight the importance of including appropriate controls, designed to enable researchers to identify and account for these differences, and harmonisation of experimental design [39, 40]. There are a number resources that support experimental design as basic guides [41] and as extensive repositories of information [42], as well comprehensive software tools, including GenEx [43], Qbase [44] and RealTime StatMiner [45].

## Accuracy and measurement uncertainty

Accuracy is essentially how close the measurement is to the truth and is influenced by both precision and bias [24]. The challenge when measuring patient samples is that the truth is often a moving target that can vary from patient to patient and within a patient, over time. The added danger with RT-qPCR is that its high precision can lead to considerable bias. This can produce results that are difficult to reproduce, either simply as a result of repeated measurements providing different estimations of the truth or, potentially worse, results that are reproducible but still biased and therefore all incorrect; this situation is problematic because agreement between laboratories leads to further confidence that the wrong result is correct.

To further understand measurement accuracy, considerations of uncertainty should be applied to indicate scientific confidence. Uncertainty has two components: systematic and random variation. Systematic errors lead to bias in the measurement. These error components are fixed and predictable and may be inherent to various instruments and methods. Random variation occurs when making repeated measurements (related to precision; a measure of the degree of agreement between replicate measurement results obtained for the same sample). Contributing factors are multitude and include issues of sampling, different analysts and each stage of the stepwise protocol necessary for a measurement [31].

The concept of accuracy includes the effect of both precision and bias and describes how close a single result is to the true value. Although it cannot be given a numerical value, measurement results are said to be ‘more accurate’ when measurement errors are reduced. Results with a small bias that are also very precise are considered highly accurate; that is, the average result is close to the true value and the data spread (standard deviation) is small. Equally, methods generating data with a large bias (large difference between the true value and the average value of the results), or imprecision (large variance), or both, would be considered inaccurate.

RT-qPCR is typically performed either by estimating copy number using a calibration curve or by simply assessing the fold change without considering the absolute abundance of the respective RNAs; the latter is termed the Δ*C*_q_ (or Δ*C*_t_) method [46, 47]. Considerations around what is accurate differ between the two methods. The former has the added challenge of how appropriate and transmutable the choice of calibrator is. A calibration curve provides an estimation of the magnitude and dynamic range of a given measurement, but can reduce or increase bias of the estimated copy number (depending on the initial value assignment).

A calibration curve also provides an estimation of the PCR efficiency, which is an important source of bias when estimating both copy number and fold change. Consequently, although the Δ*C*_q_ method ignores magnitude, PCR efficiency should ideally be estimated [47] to avoid biases. Where PCR efficiency is not routinely estimated, which is commonest for the Δ*C*_*q*_ method [46], biases could be avoided by factoring in additional uncertainty to account for the unknown PCR efficiency. This would reduce the chance of measuring a significant difference, but increase the chance that when a difference is significant, it is real.

The evaluation of background-normalised qPCR data can be subjective; for example, assessing the quality of a curve, or determining the perfect starting point of the exponential phase and where to assign the threshold for *C*_q_ generation. These elements are subject to personal judgment. For this reason, digital PCR is seen as a promising alternative, where a digital output is produced (presence or absence of target) [16, 48] and the ambiguity associated with *C*_q_ measurement is negated. For RT-qPCR measurements, calibration-curve-estimated copy number or fold changes should be reported rather than *C*_q_, which is an arbitrary measure, and assay efficiency should ideally be taken into account.

Experimental replication serves to improve confidence as it provides a better estimation of the mean provided by a given technique. Nevertheless, replication cannot assist where systematic errors are present, and may serve to make matters worse by increasing the confidence in the biased result. For RNA measurement, bias can be reduced by aiming to replicate the experimental steps that afford the highest variance from sample to analysis. This will reduce precision, but will also reduce bias. Another essential method for reducing measurement uncertainty is to apply normalisation.

## The use of normalisation and reference genes

Normalisation is an essential component of a precise mRNA measurement. Its purpose is to remove technical error. However, as with the measurement of the genes of interest, normalisation strategies are also influenced by variance and bias, and so must be used with caution. Current normalisation methods include standardising tissue weight, tissue volume, cell count and RNA concentration, or using reference genes and external reference panels [39, 49–51]. A standard approach relies on reducing gross variation by ensuring samples are of comparable size, with subtler variation (crucial to fine measurements) being further removed using (preferably multiple) internal reference genes, and/or synthetic internal positive controls.

Challenges associated with representative sampling of clinical samples are discussed in detail in the following sections, but ensuring samples are comparable can be a further challenge. Under controlled conditions of reproducibly extracted, good-quality RNA, initial gene transcript number is ideally standardised to cell number, but accurate enumeration of cells is often precluded when starting with solid tissue [49]. Another frequently applied normalisation scalar is RNA concentration. Following RNA extraction, the quantity and quality of extracts may be measured [26, 39, 52].

### Normalisation to RNA quantity

There are a number of methods for RNA quantification. Fluorescent nucleic acid binding dyes, such as RiboGreen (Life Technologies), exploit the increase in fluorescence seen on association with RNA. The reagent literature states that RiboGreen does not detect significant sample contamination by free nucleotides and thus more accurately measures the amount of intact RNA in potentially degraded samples than *A*_260_. Measuring the absorbance at 260 nm using spectrophotometry is a common and simple method for RNA quantification. Studies have shown both *A*_260_ and RiboGreen analysis methods generate comparable results when the RNA concentration exceeds a minimum of 100 ng/μL. Although *A*_260_ analysis becomes less reliable at lower RNA concentrations [32], it should be remembered that methods that use fluorescent dyes typically require a calibration curve and that the calibrator used for this must also be assigned a value (usually by *A*_260_ measurement). As with any measurement, these approaches have their own inaccuracies when used for estimating nucleic acid concentration [32, 53–57].

When using RNA concentration for normalisation, RNA quality is also an important consideration. Methods for estimating RNA quality based primarily on the detection of ribosomal RNAs (rRNAs) are very popular. Agarose gels or ‘lab-on-a-chip’-based capillary electrophoresis platforms allow RNA sample quality assessment, with the latter also offering an estimation of quantity [32]. Ribosomal RNA (rRNA) ratios, with additional electrophoretic trace features, are used to calculate total RNA integrity (e.g. RNA integrity number and RNA quality indicator). However, it should be noted that rRNAs yielding similar RNA integrity numbers/RNA quality indicators generated by these instruments can contain mRNAs that differ significantly in their integrity [58], so good-quality rRNA is not necessarily indicative of good-quality mRNA. In some instances it is impossible to quantify this parameter; for example, when minimal RNA is available from microdissected tissues [49]. A further drawback to the use of 18S or 28S rRNA molecules as standards is their absence in purified mRNA samples.

### Normalisation to reference genes

RT-qPCR analysis of mRNA should also be normalised using internal reference genes. Their suitability must be validated experimentally for particular tissues or cell types on an experimental-specific basis [59]. Ideally, normalisation should be performed against validated multiple reference genes. Further support for reference gene selection may be found using algorithms such as geNorm [49], NormFinder [60] or BestKeeper [61]. In general, using fewer than three reference genes is not advisable [25, 49, 62–64]. Single reference genes may be used if the measurement of small differences is not necessary, but the target chosen must be validated across the range of experimental conditions under investigation [65]. Crucially, any difference that is measured would need to be sufficiently greater than the inherent variation of the single reference gene measurements (incorporating all the steps from sampling to measurement) used to normalise the data, to be sure the observation is due to the gene of interest and not due to the reference gene or a combination of both.

It is increasingly evident that a number of classically designated reference genes demonstrate inconsistent expression between different tissues and treatment regimens [25, 49, 50, 59, 64, 66, 67]. For example, despite continuing reports for more than a decade that emphasise the problems associated with its use, glyceraldehyde 3-phosphate dehydrogenase continues to be used as a normaliser [32, 68, 69]. It is well documented that glyceraldehyde 3-phosphate dehydrogenase mRNA levels are not always constant [63, 67, 70], and it contributes to diverse cellular functions, such as nuclear RNA export, DNA replication, DNA repair, exocytotic membrane fusion, cytoskeletal organisation and phosphotransferase activity [71]. Although contemporary publications still fail to use appropriate reference gene(s), since the publication of the MIQE guidelines there has been an escalation in the number of publications directly evaluating reference gene validation [66, 72–74].

A recently described alternative normalisation technique targets expressed repetitive elements (expressed *Alu* repeats) [58] that are abundant in the human genome (approximately one million copies). This strategy uses *Alu* repeat sequences embedded in the untranslated regions of mRNAs, to estimate the global mRNA quantity. As a result, it has the potential to be used as a ‘universal’ internal target; that is, it is suitable to use for normalisation in all human RT-qPCR experiments. However, further work is needed to assess the validity of this proposed method.

## Other sources of bias: extraction and inhibition

There are several sources of bias in an RNA measurement by RT-qPCR; the main causes are summarised in Table 1. The presence of inhibitors has the potential to increase measurement bias, reduce assay sensitivity and produce false-negative results in both RT-qPCR and reverse transcription qualitative PCR assays. Inhibitors can come from many sources, including co-purified cellular or tissue components, carry-over components from storage buffers and the extraction process, and the reverse transcription reaction. For example, biological samples from different sources (human plasma from two different patients) may comprise distinct protein profiles. The inconsistency between these different ‘background matrices’ may alternately influence experimental outcomes due to differential target recovery and co-purified inhibition effects. Furthermore, calibration curves that are prepared in a reaction that is not affected by the inhibitor may yield biases. Recognising the importance of matrix-specific standards helps to identify the influence of the sample matrix on the accuracy of analytical results [75] and ensures that temporally separated measurements may be compared meaningfully.

**Table 1:** Factors contributing bias to a reverse transcription quantitative PCR (RT-qPCR) measurement

Source of bias	Details	Solution
RNA extraction	Poor extraction efficiency. Limited amount of RNA available. Bias towards more abundant targets, with minority species potentially measured as absent	Optimise extraction process by comparing different procedures
RNA quality	RNA degradation will lead to a reduced abundance of mRNA species. Biased cDNA production and reduced detection sensitivity, with some species being measured as absent. May affect some targets more than others	Avoid multiple freeze–thaw cycles; use of multiple aliquots of RNA/cDNA is cumbersome but essential to reduce the impact of freeze–thaw. Use RNase/DNase-free plastics and RNase decontaminating solutions and sprays and molecular grade distilled water. Change gloves frequently. Estimate sample quality where possible. Consider multiple measurements of mRNA at different locations on transcript
RNA/cDNA storage	RNA/cDNA degrades over time	Empirically evaluate stability of RNA under the storage conditions during the study period
Non-linearity of method	Caused by inhibition, enzyme inefficiency (e.g. resulting in not all RNA being converted to cDNA in the reverse transcription reaction), etc	Validate reverse transcriptase for sample type. Include appropriate controls. Aim not to add too little/too much RNA or widely differing amounts of RNA in reverse transcription reactions
Inappropriate calibrator	For example, DNA standard is used when measuring RNA. Calibrator prepared in different background material/matrix to unknown samples	Where possible, ensure that calibrators are validated as appropriate for sample type and sample matrices are spiked with them
Instrument	Reverse transcription reactions and PCRs performed with differing efficiency at different positions on the thermocycler owing to variations in temperature, ramping and thermal overshoot	Ensure instrument maintenance and calibration is up to date. Rearrange distribution of samples when performing replicate experiments (for both reverse transcription and PCR) by performing plate randomisation
Pipettes	Poorly calibrated pipettes can lead to considerable systematic bias	Routine calibration of pipettes is essential. Where accuracy is paramount, gravimetric dilution will further reduce systematic bias, even with calibrated pipettes

*cDNA* complementary DNA, *mRNA* messenger RNA

Studies show that PCR inhibition can be assay-specific, with an inhibitor completely inhibiting one assay but having no effect on another [76], so where internal positive controls are used they need to be representative of the targets of interest. The SPUD assay has been developed to estimate the extent of qPCR inhibition by measuring an external spike-in from potato (*Solanum tuberosum*) in control (water) versus target samples [77]. This can be applied as DNA or RNA [78]. Analysis of *C*_q_ and assay efficiency between control and target samples for the SPUD assay indicates the extent of matrix inhibition [16, 77]. Another simple method for evaluating inhibition is to perform a serial dilution of the sample of interest. A reduced Δ*C*_q_ at higher concentrations is suggestive of reversible inhibition.

External positive controls can be used more extensively to evaluate biases associated with the extraction step. In clinical virological load monitoring, control viruses can be added to the sample prior to extraction [79, 80]. Extraction methods can purify different amounts of template with different variances, so this is an important step to replicate [81, 82]. Quantifying total RNA is a simple method for controlling for varying yields when measuring mRNA, with the accepted potential problems discussed above. However, if further rigour is required, then external RNA standards can be used. An example of such a resource is the External RNA Controls Consortium panel of synthetic RNA oligonucleotides, which has been developed for this purpose [83].

Although external RNA standards added to biological samples may provide an assessment of the variability within the proceeding experimental steps, they cannot account for any variability upstream (e.g. sampling or cell lysis). Also, purified RNAs may not always be compatible with a given extraction method. Consequently, application of external standards needs to be validated empirically, and a combined approach in conjunction with validated reference genes may be most effectual.

## Clinical measurement

RT-qPCR is an important tool that may assist in the understanding of the molecular events underlying human diseases, but it also offers a method for measuring biomarkers for the identification and stratification of a range of diseases [32, 84]. Studies have reported applying RT-qPCR for the identification of micrometastases or minimal residual disease in colorectal cancer [85], neuroblastoma [86], prostate cancer [87] and leukaemia [88]. It has been used to distinguish different types of lymphoma [89], for the analysis of cellular immune responses in the peripheral blood [90, 91], for the detection of bacterial [92] and viral [93] RNA signatures in clinical samples and for monitoring the response of human cancer to treatment [13]. Other clinically relevant applications include its use for the analysis of tissue-specific gene expression [94], identifying cytokine gene expression on ex vivo stimulation of peripheral blood mononuclear cells [95] and for cytokine mRNA profiling [96]. Novel gene expression approaches are constantly being evaluated for diagnostic purposes for numerous human diseases.

These developments may ultimately lead to the implementation of truly personalised medicine, whereby the course of treatment chosen, the response and the prognosis may centre on molecular measurements. Yet what is ominous is that despite the vast amount of published clinical research using RT-qPCR to measure putative mRNA biomarkers, few tests have as yet been transferred to the clinic for routine use. Where RNA measurements are routinely used, such as monitoring viral loads in disease states or response to a particular treatment regime, the measurements made at the beginning of the study must be compatible with those made at the end; that is, the measurement standards used to calibrate them must have long-term stability [6]. These considerations apply equally to gene expression biomarkers and collectively contribute to measurable improvements in the quality of analytical results.

In terms of clinical measurement, different capabilities may be required depending on the measurement need. For example, viral load and specific gene signatures, such as the *BCR*–*ABL* fusion transcript, require differentiation between gross changes of the target, whereas cellular gene expression is subtler and much more challenging to measure reproducibly. For example, when measuring HIV viral load, clinicians work on orders of magnitude (log_10_ scale), whereas research that measures normalised gene expression by RT-qPCR frequently presents much smaller significant differences (e.g. often less than threefold).

### Biological variability

Biological variability is one of the principal unknown entities in terms of the aforementioned considerations and represents the final factor determining whether a given RNA measurement will be of clinical value; that is, once the technical factors have been resolved, the measurement is still dependent on biology. Previously, it was assumed that the findings of randomised controlled trials were applicable to all patients. However, it is becoming increasingly apparent that this is not the case [97, 98]. Treatment outcomes as well as disease progression and manifestation have been shown to differ between patient groups, with women and ethnic minorities being under-represented in vascular surgery randomised controlled trials [99], and to depend on patient chronotype and its relationship with cancer treatment schedules [100]. The underlying cause for these findings will be due to physiological differences, many of which will manifest themselves in the gene expression profiles, suggesting that many putative surrogate mRNA biomarkers are likely to be similarly variable between different patient groups.

Gene expression profiles may change on a cyclical basis, influenced by circadian rhythms, growth and development, and other environmental factors such as stress, sustenance/nutrition, physical activity and infection, in conjunction with variability attributable to gender, race, age and time of sample collection, to name a few. These factors must additionally be considered over and above general experimental issues such as the choice of procedure, sources of error and sample contamination, in order to select a useful biomarker that can yield reproducible results. Unpicking the sources of biological versus technical variance is a crucial yet frequently neglected step in translating a measurement to the clinic.

### Sample source and storage

RNA storage and isolation must be performed to ensure both RNA integrity and removal of contaminating nucleases, genomic DNA, and reverse transcriptase or PCR inhibitors. This can be a problem with any sample source, but clinical samples are of special concern because of their complexity, and potential inconsistencies in sample size, collection, storage, and transport can lead to variable quality of RNA templates [5]. The mRNA used for clinical diagnostics and research may be derived from various tissues, obtained from biopsies, lumbar puncture, blood, urine or buccal swabs, each posing their own challenges for accurate measurement. In each case, the limitations of sample handling in real-life clinical situations will be different. It is well known that RNA is sensitive to degradation by postmortem processes and inadequate sample handling or storage [84, 101].

The sample source is a major contributor to measurement variation. RNA extractions and subsequent analyses performed from whole-tissue biopsy samples with little regard for the different cell types contained within that sample inevitably result in the averaging of the expression of different cell types, and the expression profile of a specific cell type may be masked, lost or ascribed to and dismissed as an incorrect measurement [102] because of the bulk of the surrounding cells [32, 103]. When one is working with versatile tissues such as blood, the cell number and composition may differ between two samples (even from the same patient); consequently blood volume may not be an appropriate metric to begin with, and separation of the different cell types is often performed. However, it should be remembered that any processing of live cells will impact on the cellular physiology and may directly alter the expression of the genes of interest.

Cellular separation is more difficult to achieve when analysing solid tissue samples, but may be important as significant differences have been detected in the gene expression profiles between microdissected and bulk tissue samples [103, 104]. This is particularly relevant when comparing gene expression profiles in complex tissue with multiple, phenotypically distinct cell types, within a given tumour or between normal and cancer tissue where phenotypically normal cells adjacent to a tumour may exhibit altered gene expression profiles owing to their proximity to the tumour [32, 105]. It may be possible to alleviate these pressures of sample source/cell type by performing single-cell analysis. This rapidly growing field has much to offer, but also comes with a multitude of unique challenges associated with sample processing, low mRNA abundance and data normalisation [106–109]. It should also be remembered that cell sizes may differ between different samples (such as tumour biopsy samples or where tissues are undergoing hypertrophy as part of normal physiological processes), which adds an additional challenge to data interpretation.

## Practical clinical challenges

In certain clinical situations, for example where surgical sampling is required, some of the points detailed here will reflect a utopian view that will not be practical to implement. For instance, tissue-sampling methods may differ among institutes and even among individuals within the same institution. This can be very challenging to standardise with respect to the time span of surgery, how long it takes for a sample to be fixed or frozen, etc. To ensure data comparability and increased clinical impact within such challenging circumstances, it is crucial that such conditions are defined as accurately as possible and the associated limitations are fully considered within the discussions around a given finding.

A particular mRNA result may only be possible under a very specific sampling procedure that is not easily repeatable (owing to specialist skill and/or equipment). Such findings may reveal new biological mechanisms, but unless they can be corroborated, they will be of questionable value. An example by which this can be performed could be that the samples are re-analysed (ideally including re-extraction) by a different laboratory to confirm the measurement. However, such analysis may never be translated to routine clinical care as biomarkers, and as mRNAs are frequently measured as surrogates for protein-driven physiology, additional confirmatory experiments considering the proteins and/or physiology in question are essential.

It is also crucial that other factors within the protocol (Fig. **1**) that can be controlled are detailed within a given study. Factors that frequently vary but which are easily controlled, and easily reported, such as storage conditions and duration, may differ among laboratories (e.g. type of freezer, storage in liquid nitrogen by immersion or by vapour phase), and so they must be comprehensively described. Documentation of such factors will facilitate identification of any associated discrepancies that might arise, a fact that is particularly pertinent to biobanking, which may comprise large numbers of samples that may have been stored for different durations.

## Conclusion

Accurate RT-qPCR analysis could improve clinical diagnosis as well as predictive and prognostic monitoring. Furthermore, improved analytical measurement sensitivity may offer tools to detect and quantify disease markers at earlier stages of progression, facilitating more timely treatment and improved outcome. Moreover, diagnostic tests conferring superior accuracy and analytical confidence may change the treatment regimens patients are offered. For example, therapies may be effective in treating only certain tumour genotypes and may have serious contraindications. As such, they are offered only to those patients for whom there is definitive molecular proof that they harbour the associated specific mutation. Human epidermal growth factor 2 status in breast cancer is one such example and is used as a predictive therapy-selection factor for the humanised monoclonal antibody trastuzumab (Herceptin, Genentech) [110]. Current diagnostic methods, including fluorescent in situ hybridisation and immunohistochemistry, can be subjective and insensitive. Advances in accurate molecular quantification of RNA [111, 112] could offer enhanced analytical power for this and many similar clinical challenges, and may in the future become gold standards in clinical diagnostics. RT-qPCR currently offers a powerful tool both to identify and to translate the clinical use of such biomarkers.

Yet for preclinical research using RT-qPCR to make a translatable impact, accuracy must be seen as more than just good precision. Accurate clinical measurement must also include considerations of both potential bias and good technical reproducibility. By application of this notion to the whole stepwise process from sampling to preparing the RNA sample and subsequent methods for normalisation, RT-qPCR will become more reproducible, which in turn will improve the impact and likelihood that findings will be translated to routine clinical use. Although accomplishing all the standardisation measures detailed in this review all the time may not always be possible, particularly in preclinical research, the key is to consider sources of error so that, where possible, they can be captured in the experimental design. Such an approach will improve measurement reproducibility and the likelihood that significant findings are both real and translatable to routine clinical care.
